# Hydatid Cyst of the Adrenal Gland: A Clinical Study of Six Cases

**DOI:** 10.1100/tsw.2006.375

**Published:** 2006-04-21

**Authors:** Ali Horchani, Yassine Nouira, Kais Nouira, Haikel Bedioui, Emna Menif, Zoubeir Ben Safta

**Affiliations:** Department of Urology, La Rabta Hospital, Tunis, Tunisia; ^2^ Department of Radiology, La Rabta Hospital, Tunis, Tunisia; ^3^ Department of General Surgery, La Rabta Hospital, Tunis, Tunisia

**Keywords:** adrenal, hydatid cyst, treatment, surgery, laparoscopy

## Abstract

Hydatid cyst of the adrenal gland (HCAG) is an exceptional occurrence. We report our experience of six cases of HCAG and discuss the diagnosis and treatment of this hydatid localization. We retrospectively reviewed and analyzed the clinical files of six patients admitted to our institution from January 1990 to December 2000 for HCAG. Patients varied in age from 24—59 years. They were five males and one female. One patient had a history of pulmonary hydatidosis treated surgically 10 years previously. Five patients presented with lumbar pain and one patient had bouts of hypertension, headache, and palpitation. Physical examination was normal except in one patient who was hypertensive. Preoperative diagnosis was highly suggested by ultrasonography. CT scan performed in all cases clearly showed the relationship of the cyst with adjacent organs. Serology tests were positive in two cases. One patient had elevated urine VMA and was operated on with the diagnosis of cystic phaeochromocytoma. All six patients were operated on and had either an adrenalectomy (two cases) or partial pericystectomy (four cases). In one case, partial pericystectomy was conducted through a retroperitoneal laparoscopic approach. The hydatid nature of the cyst was confirmed pathologically. All patients had a smooth postoperative course with no cystic recurrence on follow-up. The diagnosis of HCAG is based mainly on ultrasonography and CT scan. Surgery with either partial or total excision of the cyst, with or without preservation of the adrenal gland, is the treatment of choice.

## INTRODUCTION

Cystic echinococcosis or hydatid disease is a parasitic infection caused by a tapeworm, *Echinococcus granulosus*, that forms larval cysts in human tissues[17].

Hydatid disease is endemic in many pastoral and farming regions of the world[5]. Infestation with echinococcus is quite common in Tunisia with an incidence estimated to 11.3/100,000 inhabitants[2].

Although cysts may localize at any body tissue, adrenal glands are rarely involved and account for less than 0.05% of all hydatid localizations[7].

Herein, we report our experience of six cases of hydatid cysts of the adrenal gland (HCAG) admitted to our institution from January 1990 to December 2000 and discuss the diagnosis and treatment of this hydatid localization.

## PATIENTS AND METHODS

The records of all patients operated on in the departments of Urology and General Surgery of La Rabta Hospital, Tunis, Tunisia, between January 1990 and December 2000, were reviewed. During this 11-year-period, six patients were operated on for HCAG. One case was previously reported as a separate case report[5]. These six cases are summarized in Table 1.

| Patient | Age | Gender | History | Side | Size (mm) | Symptoms | Symptom duration | Serology | Ultrasound | CT scan | Treatment |
|---|---|---|---|---|---|---|---|---|---|---|---|
| **1** | 24 | M | No | R | 56 | Dull flank pain | 6 months | Positive | Proper-walled cystic mass | Cyst + tiny peripheral calcifications | Transperitoneal subcostal partial pericystectomy |
| **2** | 47 | M | No | L | 80 | Dull flank pain | 3 months | Negative | Proper-walled cystic lesion | Cyst + calcified wall | Retroperitoneal laparoscopic partial pericystectomy |
| **3** | 55 | M | No | R | 60 | Flank pain + headache + palpitation + hypertension | 6 months | Negative | Heterogeneous cystic mass with multiple vesicles | Multicystic mass with peripheral calcifications | Transperitoneal subcostal adrenalectomy |
| **4** | 59 | M | Lung hydatid cyst operated | L | 70 | Dull flank pain | 6 months | Positive | Proper-walled cystic lesion | Heterogeneous cyst | Lumbar incision partial pericystectomy |
| **5** | 54 | M | No | L | 60 | Dull flank pain | 2 months | Negative | Proper-walled cystic lesion | Cyst + tiny peripheral calcifications | Transperitoneal subcostal adrenalectomy |
| **6** | 44 | F | No | R | 50 | Pain in right upper abdominal quadrant | 9 months | Negative | Cystic lesion with calcified wall | Cyst + punctuate peripheral calcifications | Transperitoneal subcostal partial pericystectomy |

**Table I:** Summary of clinical and radiological data of our six patients

There were five men and one woman and their ages ranged from 24–59 years (median: 47 years). All six patients had unilateral cyst with no other concomitant hydatid localizations.

One patient, a 59-year-old man**,** had undergone an operation 10 years previously for left pulmonary hydatid cyst. All patients had plain abdominal X-ray films, ultrasound, and computed tomography (CT). Indirect hemagglutinin (IHA) test for *Echinococcus granulosus* was obtained in every case. One patient with symptoms suggestive for phaeochromocytoma had VMA and catecholamine metabolites levels measured[20].

## RESULTS

Moderate and dull flank pain was the presenting symptom in all our six patients. The IHA test was positive in only two cases. Plain abdominal X-ray films showed calcifications in the suprarenal region in two cases (Fig. 1). Ultrasonography showed a proper-walled cystic lesion of the adrenal gland in all cases (Fig. 2). Peripheral calcifications were seen in one case as well as intracystic vesicles (daughter cysts) in one case. CT findings suggestive for hydatid cyst were a well-circumscribed cystic lesion that did not enhance after contrast medium with tiny peripheral calcifications (calcified pericyst) (Fig. 3). Presence of daughter cysts was very suggestive of hydatid origin. Definitive diagnosis of HCAG was obtained by macroscopic and pathologic examination of the cyst and its content in all six cases.

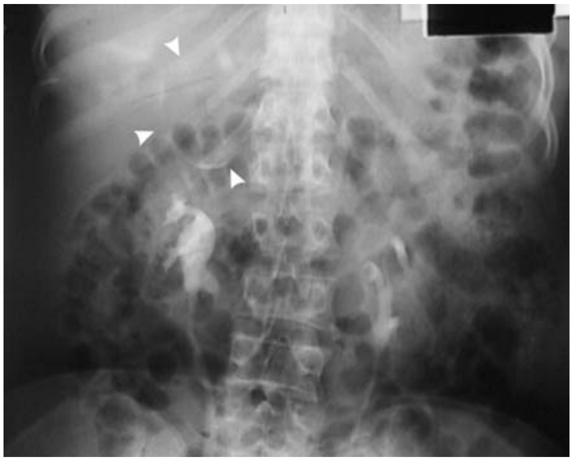

**FIGURE 1**. Intravenous urography showing curvilinear calcifications in the left suprarenal region (arrowheads).

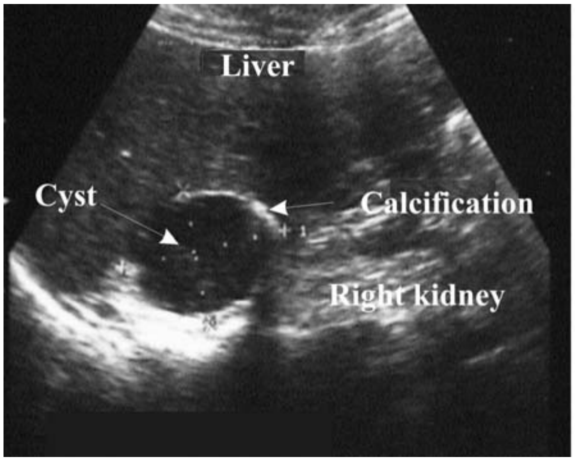

**FIGURE 2**. Ultrasonographic image showing a well-circumscribed, proper-walled calcified mass of the right adrenal gland.

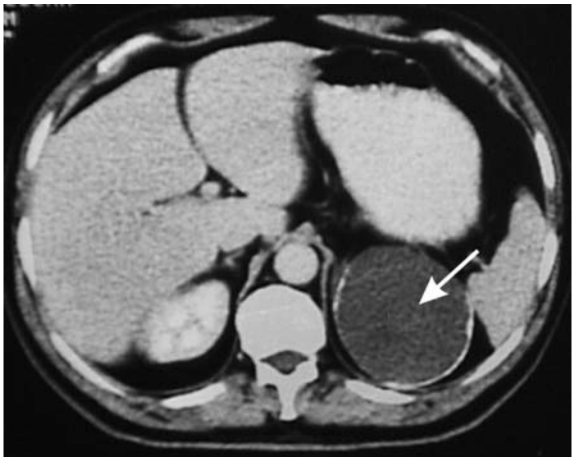

**FIGURE 3.** CT scan showing a left adrenal cystic lesion with tiny peripheral calcifications (arrow).

One patient with symptoms suggestive for a phaeochromocytoma had elevated VMA levels in two blood samples, while serum catecholamines and metabolites levels were normal.

This patient was misdiagnosed for a cystic phaeochromocytoma and had right adrenalectomy through a subcostal transperitoneal approach.

Pathological examination of the resected specimen concluded to a HCAG. Normalization of blood pressure and abatement of all symptoms occurred postoperatively.

Total adrenalectomy was performed in two cases, while in four cases only partial resection of the cyst and the surrounding pericyst (after sterilization of the cystic content with hypertonic saline) was conducted. The ipsilateral adrenal gland was preserved in these four cases.

One patient had laparoscopic retroperitoneal partial resection of the cyst with good outcome.

No patient was treated with benzimidazole compounds. Patients' postoperative courses were uneventful.

With a mean follow-up period of 2 years, no patient showed hydatid cyst recurrence.

## DISCUSSION

The adult *Echinococcus granulosus* is present in the small intestine of canine animals. The echinococcal eggs excreted in the feces of canine animals are ingested by intermediate hosts like sheep, cattle, or humans. These eggs hatch in the intestine of the intermediate hosts, penetrate the intestinal mucosa, and enter the portal circulation. Not all embryos survive, many are overcome by the host's defense mechanism. If not filtered out by the liver, embryos enter the general blood circulation and may lodge in any organ[24]. Embryos develop into hydatic cysts, the larval phase of the parasitic cycle, with very slow growth. It takes 3–6 years for the development of a cyst the size of a hen's egg[9]. The host reacts against the parasite with a pericyst of fibrous and connective cyst elements.

Adrenal cysts are rare, with an incidence at autopsy of 0.06–0.18%[14,23]. Hydatid cyst of the adrenal gland is exceptional and accounts for only 6–7% of all adrenal cysts[1,14,23], but in endemic areas, most of the adrenal cysts requiring operations are of hydatid nature. Out of 15 patients diagnosed with adrenal cyst in the series of Akçay et al.[3], 9 were hydatid cysts. Derived from the classification of Foster[14], other adrenal cysts include: (1) true cysts, the most common of whom are endothelial cysts mainly hemangiomas and lymphangiomas (45%) and epithelial cysts such as glandular retention cysts and embryonic cysts (9%); and (2) pseudocysts caused by hemorrhage (39%)[8].

Most of the time, adrenal cysts are asymptomatic and diagnosed at autopsy[14] or incidentally on imaging studies[3]; however, patients may present with vague, nonspecific symptoms.

The most prominent clinical features consist of dull pain in the renal area[3]. Exceptional HCAG causes hypertension, which is described as Goldblatt phenomenon[15,25]. This can be explained by irritation of the functional tissue of the adrenal gland by the growing cyst[15].

Hypertension is usually curable by the cyst ablation as was the case in one of our patients. Anaphylactic shock can result from the rupture of hydatid cyst[23] and is considered a serious complication of hydatid disease.

Serology is useful in the diagnosis of hydatid disease. However, the sensitivity of serology tests is affected by the site and condition of hydatid cysts in the body[10]. Hydatid cysts in human lungs, spleen, or kidneys tend to be associated with lower serum antibody levels compared to the liver[21]. In our series, only two patients out of six had positive serology tests. Degenerating cysts are associated to low serum antibody levels[17]. Interpretation of hydatid serology should be cautious and confronted to radiological findings. Ultrasonography is the key diagnostic tool in cases of hydatid disease. Its diagnostic sensitivity in abdominal echinococcosis ranges from 93–98%[6]. In our series, ultrasonography was suggestive of hydatid cyst in five out of six patients by showing a proper-walled cystic lesion. Presence of other cysts at various stages of evolution in the liver or other organs makes interpretation easier.

Although many authors think that parasitic involvement of the adrenals is usually secondary and part of a generalized echinococcosis[1,3,23], only one patient in our series had evidence of other hydatid localization, suggesting secondary echinococcosis.

Tiny curvilinear calcifications in the suprarenal area seen on plain radiography (due to calcium deposit in the pericyst), are suggestive of hydatid cyst[16,17], although tuberculous abscess or hemorrhagic pseudocysts may calcify[11].

On CT scan, a smooth, round, low-density, noncontrast-enhanced mass with a thin wall is very suggestive of hydatid cyst[11]. CT scans can also show peripheral linear or punctuate calcifications or peripheral small fluid collections from secondary vesicles (daughter cyst sign)[18,24].

The sensitivity of CT scan in abdominal echinococcosis is estimated to 97%[11]. MRI proved not to be cost effective in the diagnosis of hydatid disease[19] and, in our opinion, is not justified in the investigation of hydatidosis.

Although CT-guided needle aspiration may be useful for diagnosing adrenal cystic lesions, this is not indicated when a hydatid cyst is suspected because of the high risk of hydatid dissemination[12] and anaphylactic shock[4].

Treatment of HCAG is surgical and implies treatment of the cyst with a scolicide and the remaining cystic cavity.

Ideally, total cyst excision is conducted; however, when the cyst is large and adherent to adjacent structures that need to be preserved, partial pericystectomy and adequate drainage of the remaining cystic cavity is an acceptable alternative.

Total adrenalectomy is not mandatory and is performed when the cyst has almost completely destroyed the gland[6,13]. In our series, four patients had partial or total cystectomy and two had adrenalectomy.

The surgical approach through a laparotomy incision has the advantage of allowing the surgeon to explore the peritoneal cavity and search for other hydatid localizations overlooked by imaging techniques. Laparoscopic resection of an adrenal hydatid cyst can also be an adequate procedure[11] and was used in one case of our series. It is of utmost importance to inactivate the cyst before manipulating it. Prior to dissection, a scolicidal agent such as hypertonic saline is injected inside the cyst in order to destroy daughter cysts and scolices, and to avoid spillage of viable cystic content resulting in secondary echinococcosis with implantation of parasites in the peritoneal and retroperitoneal regions.

Chemotherapy with benzimidazole compounds (albendazole and mebendazole) in hydatid disease is of questionable value and should be reserved for inoperable patients and those with disseminated lesions[22].

## CONCLUSIONS

Hydatid cyst of the adrenal gland is an exceptional location that should be kept in mind and thought of in endemic areas or in patients with a history of hydatid disease in other organs.

Diagnosis is based mainly on ultrasonography and CT. Surgical total or partial excision of the cyst with adrenal preservation is the treatment of choice.
